# Stenting for a Ruptured Blood Blister-Like Aneurysm of the Basilar Artery: Case Report and Literature Review

**DOI:** 10.7759/cureus.84353

**Published:** 2025-05-18

**Authors:** Tadahiro Ishiwada, Mariko Noda, Shogo Imae, Osamu Tone

**Affiliations:** 1 Department of Neurosurgery, Fujiyoshida Municipal Hospital, Fujiyoshida, JPN; 2 Department of Neurosurgery, Yamato Tokushukai Hospital, Yamato, JPN

**Keywords:** basilar artery, blood blister-like aneurysm, endovascular treatment, stenting, subarachnoid hemorrhage

## Abstract

Blood blister-like aneurysms (BBAs) usually occur on the supraclinoid portion of the internal carotid artery. There are a few reports of BBAs in other locations. We provide the case details of a ruptured BBA of the basilar artery (BA) that was treated with stenting, with a good outcome. The patient was a 74-year-old Japanese woman with a World Federation of Neurosurgical Societies (WFNS) grade I subarachnoid hemorrhage. Digital subtraction angiography (DSA) performed on day 14 revealed a BBA with a maximum diameter of 1.5 mm and a wide neck on the posterior wall of the BA's tip. Single stenting was performed on day 20, and an additional stenting was performed on day 26 due to postoperative stent migration. At six months post-operation, DSA revealed that the aneurysm had disappeared, and the patient was recovering well. Ruptured BBAs of the BA are rare and difficult to treat due to high rates of rebleeding and the presence of significant perforating branches. BBAs located on the posterior wall, in particular, are at high risk of ischemic complications. If coil embolization within an aneurysm is difficult, stenting alone may also be an effective treatment.

## Introduction

A blood blister-like aneurysm (BBA) is a small, hemispherical bulge from the nonbranching site of the cerebral artery. BBAs account for 0.5%-2% of intracranial ruptured cerebral aneurysms [1]. They are thought to be caused by a small laceration of the internal elastic lamina, but there is no consensus on the mechanism. In BBA cases, the vessel wall is locally defective and covered with thin fibrous tissue, and it has a form similar to a pseudo-aneurysm; the risk of rebleeding is thus extremely high [2]. It is difficult for neurovascular surgeons to treat BBAs because of the fragility of the vessel wall and the indistinct neck. BBAs usually occur on the supraclinoid portion of the internal carotid artery (ICA), but they have been observed at sites throughout the cerebrovascular system [3,4]. BBAs occurring in the basilar artery (BA) are extremely rare and strictly distinct from basilar perforator artery aneurysms. Due to the anatomical characteristics of the BA's region and the proximity of perforating branches, the treatment of a BBA in the BA is even more complicated [5-7]. Open surgery and endovascular treatment are available options for this lesion, but the best treatment has not been established. We describe the case of a ruptured BBA of the BA that was treated with stenting, with a good outcome.

## Case presentation

The patient was a 74-year-old Japanese woman. She was brought to a hospital by ambulance after the sudden onset of neck pain and vomiting while she was stretching. She had a history of hypertension and dyslipidemia and was being treated for these conditions with medication. Upon her arrival at the hospital, her blood pressure was 168/89 mmHg and her pulse rate was 91 beats per minute. Her level of consciousness was Glasgow Coma Scale (GCS) score E4V5M6 and World Federation of Neurosurgical Societies (WFNS) grade I, which is the mildest condition. There was no paralysis or other neurological deficits.

**Figure 1:**
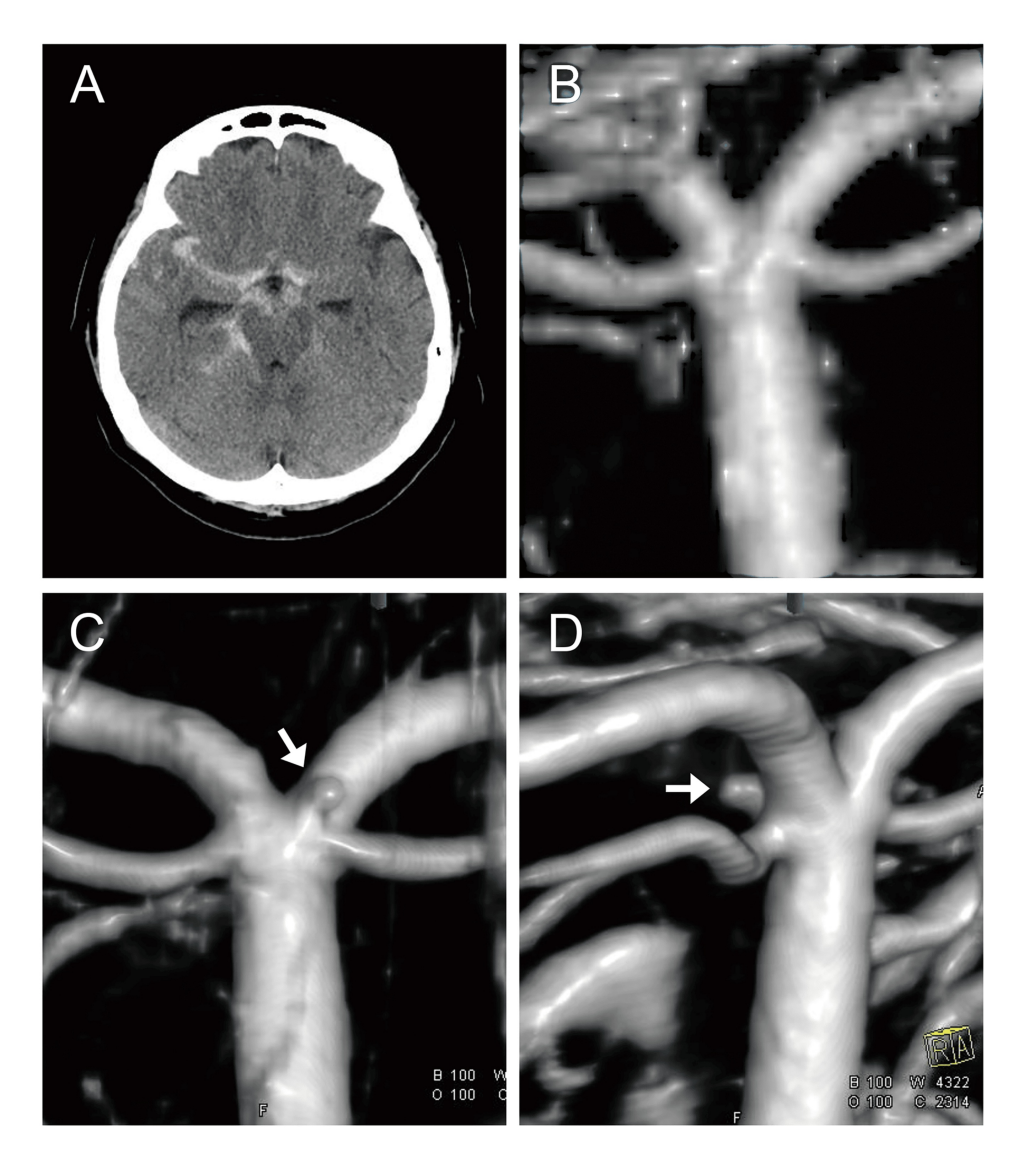
Imaging findings for the patient, a 74-year-old woman. A: Initial head CT scan showed a subarachnoid hemorrhage in the right prepontine cistern and right Sylvian fissure. B: 3D-DSA performed on day 2 showed no obvious aneurysm. C, D: 3D-DSA performed on day 14 showed a blood blister-like aneurysm on the posterior wall of the basilar artery tip (C: posterior view, D: right anterior oblique view). Arrows indicate the aneurysm. DSA: digital subtraction angiography.

A CT scan of the patient's head revealed a subarachnoid hemorrhage (SAH) in the right prepontine cistern and right Sylvian fissure (Figure 1A). Head computed tomography angiography (CTA) showed no cerebral aneurysms or vascular dissections. Sedation and antihypertensive treatment were administered in the intensive care unit. Digital subtraction angiography (DSA) was performed on day 2 of the patient's admission, but it did not show an obvious aneurysm (Figure 1B). With repeated CTA and DSA examinations, an aneurysm measuring 1.5 mm in maximum diameter with a wide (1 mm) neck was observed on the posterior wall of the tip of the BA on the DSA performed on day 14 (Figures 1C, 1D). This aneurysm originated from the nonbranching site of the perforating artery, so it was diagnosed as a BBA rather than a perforator artery aneurysm.

Treatment strategy

The cause of the patient's SAH was diagnosed as a ruptured BBA on the BA's tip. Surgical treatment was considered necessary to prevent rebleeding. As a treatment strategy, we considered that clipping under a craniotomy would be difficult to approach and would also pose a high risk of intraoperative rupture. Concerning endovascular treatment, we considered that stent-assisted coiling (SAC) embolization would be necessary because the aneurysm had a wide neck. In addition, because the aneurysm was very small, there was a possibility that coil placement would be difficult, and in this patient's case, we decided to do stenting only. We explained the off-label use of the stent to the patient and her family, obtained informed consent, and performed the endovascular treatment on day 20.

Endovascular treatment

**Figure 2:**
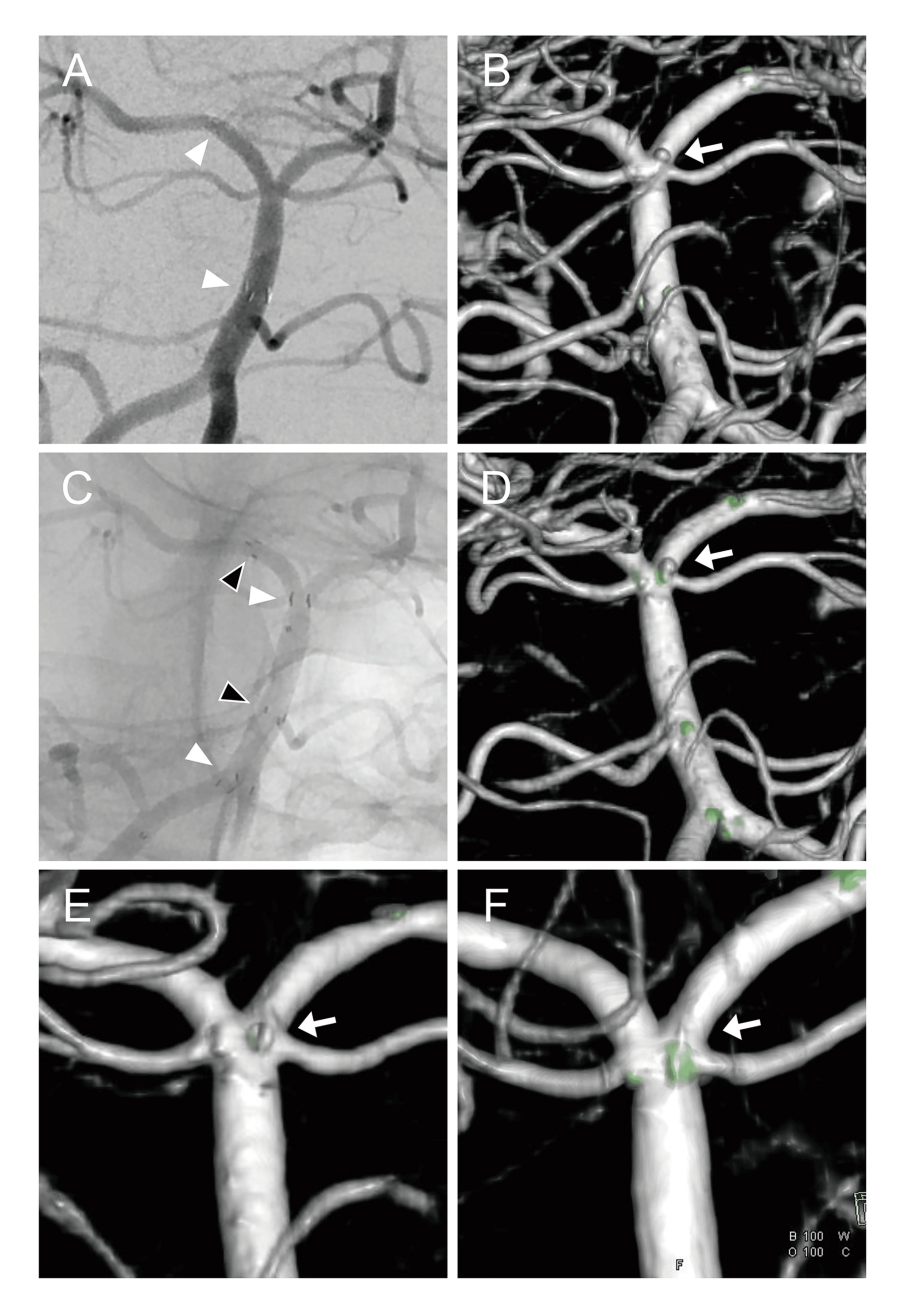
Imaging findings of endovascular treatment. A: Angiography (anterior view) after initial treatment. An Enterprise2 stent was deployed from the right posterior cerebral artery to the basilar artery (BA) (white arrowheads). B: 3D-DSA (posterior view) after initial treatment showed that blood flow remained in the aneurysm (arrow). C: Angiography (anterior view) after the second treatment. The Enterprise2 stent had migrated into the BA (white arrowheads). A Neuroform Atlas stent was deployed from the right posterior cerebral artery to the BA (black arrowheads), overlapping the Enterprise2. D: 3D-DSA (posterior view) after the second treatment showed that blood flow remained in the aneurysm (arrow). E: 3D-DSA (posterior view) one month after the treatment showed that the aneurysm was unclear (arrow). F: 3D-DSA (posterior view) six months after the patient's treatment demonstrated that the aneurysm had disappeared (arrow). DSA: digital subtraction angiography.

Dual antiplatelet therapy, i.e., 200 mg of aspirin and 150 mg of clopidogrel, was administered orally as a loading dose starting the day before the patient's surgery. Under general anesthesia, a 5Fr Envoy catheter (Johnson & Johnson, New Brunswick, NJ, USA) was inserted through the right femoral artery into the left vertebral artery (VA). Heparin was administered. A Prowler select Plus 45° microcatheter (Johnson & Johnson) was inserted into the right posterior cerebral artery (PCA), and an Enterprise2 VRD 4.0×23-mm stent (Johnson & Johnson) was deployed from the right PCA to the BA (Figures 2A, 2B). We attempted to place an Echelon 10 90° microcatheter (Medtronic, Minneapolis, MN) into the aneurysm using the trans-cell method guided by a 0.012-inch GT Wire guidewire (Terumo, Tokyo), but this was difficult. There was also a risk of aneurysm perforation, and thus the procedure was ended with stenting only.

The day after the patient's first surgery, a CT scan showed that the stent had migrated into the BA. A second surgery was thus performed on day 26. Under general anesthesia, a 5Fr Envoy catheter was inserted through the right radial artery into the right VA. The Enterprise2 stent had migrated into the BA, and the aneurysm was barely covered. An Excelsior SL-10 preshaped 45° microcatheter (Stryker, Kalamazoo, MI) was passed through the stent and inserted into the right PCA, and a Neuroform Atlas 4.0×21-mm stent (Stryker) was deployed from the right PCA to the BA overlapping the Enterprise2 (Figures 2C, 2D). We attempted to place the SL-10 microcatheter or an Echelon 10 microcatheter in the aneurysm by using the trans-cell method guided by a 0.012-inch GT Wire guidewire, but this was also difficult. Although blood flow remained in the aneurysm, coil placement was abandoned.

Postoperative course

The patient had no neurological deficits after surgery. The patient's postoperative CT scan showed no expansion of the SAH, and the MRI indicated no appearance of cerebral infarction. DSA performed on day 63 revealed no change in the position of the two stents, and the aneurysm was unclear (Figure 2E). The patient participated in rehabilitation and was discharged home on day 71 with a modified Rankin Scale (mRS) score of 0, no symptoms at all. She continued to take 100 mg of aspirin and 75 mg of clopidogrel daily for three months after the surgery and then continued to take clopidogrel only. DSA performed six months after the surgery demonstrated that the aneurysm had disappeared (Figure 2F). More than three years has passed since the patient's surgery, and no neurological deficits have developed and the aneurysm has not recurred.

## Discussion

Treatment for blood blister-like aneurysms

A BBA is a lesion that is generally difficult to treat, as it is associated with a high rate of rebleeding. In the past, open surgical treatments such as clipping, wrapping, clip-on wrapping, and trapping with or without bypass were the main treatments for a BBA, but this resulted in a high rate of intraoperative bleeding and did not produce good results. In recent years, endovascular treatment techniques have been developed and new treatments for BBAs have become possible [8,9]. Ren et al. reported the results of 33 cases of surgical treatment and 50 cases of endovascular treatment for BBA of the ICA [8]. The proportion of patients with a good outcome at one year after treatment was 54.5% in the surgical group and 76.0% in the endovascular group, with significantly more patients in the endovascular group having a good outcome (p=0.041). Shah et al. conducted a meta-analysis comparing the results of surgical (139 procedures) and endovascular (122 procedures) treatment [9]. The immediate and short-term aneurysm occlusion rates were higher with surgical treatment. However, the incidence of complications was lower after endovascular treatment than after surgical treatment, and the functional prognosis was better.

Endovascular treatment for BBAs can be divided into deconstructive and reconstructive treatments. Deconstructive treatments include trapping and parent artery occlusion, which are reliable methods of occluding the lesion site but may not be suitable for major vessels such as the ICA. The reconstructive treatments for BBAs include coil embolization, SAC, stent placement (single or overlapping), and flow diverter (FD) placement. Because BBAs present with a small and wide neck shape, coil embolization alone is often difficult and treatment using neck bridge stents or FDs is the norm [10-12]. Rouchaud et al. reported the results of 25 cases of deconstructive treatment and 240 cases of reconstructive treatment for ruptured BBA [10]. Their deconstructive-treatment group had a higher initial occlusion rate than the reconstructive-treatment group (77.3% vs. 33.0%) but also a higher risk of perioperative ischemic stroke (29.1% vs. 5.0%). The proportion of patients with a good outcome was similar (79.9% vs. 76.2%). In addition, among the reconstructive treatments, the FD cases had a higher rate of complete occlusion in the medium to long term (90.8% vs. 67.9%) and a lower rate of retreatment (6.6% vs. 30.7%) compared to the other reconstructive treatments.

In recent years, there have been many reports of reconstructive treatment using SAC or FD. Fang et al. reviewed 213 cases of the use of SAC embolization for BBAs and reported that complete occlusion was achieved in 134 cases (64.6%) and recurrence developed in 49 cases (22.9%) [11]. The occlusion rates were higher and the recurrence rates were lower when two or more stents were used. In addition, the incidence of hemorrhagic complications was low even when two or more stents were used, and there was no significant difference in the incidence of ischemic complications. The use of two or more stents is recommended in cases in which SAC embolization is applied. Mokin et al. described 43 cases of BBA treated with FD: 38 cases (68%) had a good outcome, with one case of rebleeding after treatment [12]. Of the 32 cases that could be followed up, complete occlusion was achieved in 28 (87.5%), and there was no significant difference in the rate of complete occlusion between the use and non-use of coils. At present, reconstructive treatment using SAC embolization or an FD is probably the most appropriate treatment for BBAs.

Treatment for blood blister-like aneurysms of the basilar artery

BBAs occurring in the BA are especially difficult to treat. Because there are many important perforating branches in the BA, ischemic complications must be considered in addition to hemorrhagic complications. Ischemic complications have a high probability of leaving severe symptoms, making deconstructive treatment difficult and reconstructive treatment necessary [5-7]. BBAs occurring in the BA are extremely rare. Our search of the relevant literature identified 15 papers on the subject and a total of 32 cases, including our patient's case [1-7,13-20]. The treatment methods used were as follows: open surgery (n=7 cases), endovascular treatment (n=24), and combined treatment (n=1). Mooney et al. reported four cases of direct clipping surgery for BBAs of the BA [2]. In one case, an additional endovascular stent was required for the residual aneurysm after clipping, but complete occlusion was achieved in all aneurysms and there were no recurrences. However, the BA is a difficult vessel to approach with open surgery, and there are few reports of this strategy. The main method of treatment is endovascular reconstructive treatment.

Of the 24 reported patients whose BBAs underwent endovascular treatment, the 17 cases with detailed clinical data are summarized in Table 1. The median age of the patients was 53 years (range, 44-80 yrs), and 81% (n=13) were female. The location of the aneurysm was distal (n=9 cases), middle (n=4), and proximal (n=0), with most cases occurring in the distal portion of the BA. The anterior wall was involved in five cases, the posterior wall in seven cases, and the lateral wall in three cases. The size of the aneurysm was very small (≤3 mm) in all cases, and all of the aneurysms had a wide neck. The timing of treatment varied from one to 42 days after onset. The initial treatment included SAC embolization (n=5 cases), single stent placement (n=2), overlapping stent placement (n=4), and FD placement (n=6). Four cases (24%) required retreatment. Both of the two patients who initially underwent single stent placement required retreatment, with one patient undergoing additional stent placement and the other patient undergoing additional FD placement. Of the four patients who initially underwent overlapping stent placement, two required retreatment, with one patient undergoing additional stent placement and the other patient undergoing SAC embolization. None of the patients who initially underwent SAC embolization and FD placement required retreatment.

**Table 1:** Cases of endovascular treatment for ruptured blood blister-like aneurysm of the basilar artery. BF: body filling, CO: complete obliteration, EVD: external ventricular drain, mos.: months, mRS: modified Rankin Scale, NA: not available, NR: neck remnant, PED: pipeline embolization device, wks: weeks.

First author, year	Age, Sex	Grade	Aneurysm position	Size, mm	Timing, days	First treatment	Retreatment	Immediate angiographic result	Complications	Rebleeding	Follow-up angiographic result	Outcome, mRS
Meckel et al., 2011 [13]	49, F	4	Distal, anterior	2.1 × 4.8	6	Stent-assisted coil	None	NR	None	None	NR, 3 mos.	1
	44, F	3	Anterior	2.0 × 3.0	NA	Stent-assisted coil	None	BF	None	None	CO, 12 mos.	0
Martin et al., 2012 [14]	49, F	2	Middle, posterior	2.2 × 2.3	42	Stent (Enterprise)	Flow diverter stents (2 PEDs)	BF	Infarction of right pons	None	CO, 2 mos.	0
Lim et al., 2013 [1]	45, F	2	Middle, anterior	NA	NA	Stent-assisted coil (3 stents)	None	CO	None	None	CO, 13 mos.	0
Kim and Ko, 2014 [15]	53, M	2	Distal, posterior	2.1 × 2.0	Within 48 hrs	Overlapping stents (Neuroform, Enterprise)	None	BF	None	None	CO, 18 mos.	0
Chalouhi et al., 2014 [16]	80, F	NA	NA	3	1	Flow diverter stent (PED)	None	NA	None	None	CO, 2 wks	0–2
Aydin et al., 2015 [17]	47, F	1	Middle, right Lateral	2.0 × 2.5	15	Flow diverter stent (Silk)	None	BF	None	None	CO, 3 mos.	1
	68, NA	2	Lateral	2.5 × 4.0	8	Flow diverter stent (Silk)	None	BF	None	None	CO, 3 mos.	0
Lozupone et al., 2018 [18]	60, F	2	Distal, posterior	Small	10	Flow diverter stent (PED)	None	BF	Intraparenchymal hematoma after EVD	None	CO, 6 mos.	3
	55, F	5	NA	Small	2	Flow diverter stent (PED)	None	NA	None	None	CO, 6-12 mos.	0
Morinaga et al., 2019 [5]	52, M	4	Distal, anterior	2	10	Stent-assisted coil (2 LVIS Blue stents)	None	CO	None	None	CO, 12 mos.	3
	62, F	1	Distal, posterior	1.7	5, 14	Overlapping stents (2 LVIS Jr. stents)	Stent-assisted coil (LVIS Jr.)	BF	Infarction of right pons	None	CO, 1 week	1
Capocci et al., 2020 [19]	56, M	4	Distal, posterior	1.5	32	Flow diverter stent (PED)	None	CO	Infarction of cerebellum, thalamus and medulla oblongata	None	CO, 65 mos.	1
Yamamura et al., 2021 [6]	53, F	2	Middle, right lateral	2.5 × 4.0	1	Stent-assisted coil (LVIS Blue)	None	NR	None	None	CO, 7 mos.	0
Prabhala et al., 2021 [7]	52, F	2	Distal, anterior	1.7 × 1.6	NA	Overlapping stents (2 LVIS Jr. stents)	Stent (Enterprise)	NA	None	None	CO, 3 mos.	0
	66, F	4	Distal, posterior	1.5	NA	Overlapping stents (Neuroform, Enterprise)	None	NA	None	None	CO, 6 mos.	0
Present case	74, F	1	Distal, posterior	1.5 × 1.0	20, 26	Stent (Enterprise)	Stent (Neuroform)	BF	None	None	CO, 6 mos.	0

The immediate angiographic results were complete obliteration (CO) (n=3 cases), neck remnant (NR) (n=2), and body filling (BF) (n=8), with many cases ending in BF. Post-treatment complications were observed in four cases (24%), including three cases of cerebral infarction and one with a hemorrhagic complication associated with external ventricular drainage. Interestingly, all three cases with ischemic complications were aneurysms that occurred on the posterior wall of the BA. There were no cases of rebleeding. Follow-up angiographic results were CO in 16 cases and NR in one case, and complete occlusion was achieved in most cases (94%). The outcome was good in all cases with mRS values 0-3.

The initial treatment should be SAC embolization if coil placement can be conducted safely and an overlapping stent or FD placement if this is difficult. As single stent placement alone often requires retreatment, it is preferable to use two or more stents. FD treatment is expected to be effective in the future, but because a BBA that occurs on the posterior wall in particular is often associated with ischemic complications [5,14,19], extra care must be taken to avoid excessive metal coverage. In addition, the use of perioperative antiplatelet agents is essential, but care must also be taken to prevent hemorrhagic complications. It is necessary to accumulate more cases of BBA in the BA and to conduct sufficient studies in order to establish the optimal treatments.

Discussion of this case

The initial treatment for our patient was a single stent placement, but an additional treatment was performed with an overlapping stent placement and the aneurysm was finally completely occluded. The first stent, Enterprise, was selected to facilitate the trans-cell method and the second stent, Neuroform, was chosen to accommodate the curvature of the parent vessel. Consequently, the metal coverage rate was relatively low. There were no rebleeds or ischemic complications, and the patient's condition improved smoothly. As the aneurysm was very small, coil placement was difficult, but it might have been possible to use the jailing or jack-up method. Coil placement is an invasive procedure that poses a risk of intraoperative rupture. In addition, as our patient's aneurysm was on the posterior wall, there was also a risk of ischemic complications from coil placement. If coil embolization within an aneurysm is difficult, stenting alone may also be an effective treatment, but it may be necessary to use two or more stents to achieve aneurysm occlusion. In the future, FD treatment is expected to be effective in similar cases.

Study limitations

The use of neck bridge stents and FDs in the acute phase of a rupture is not covered by insurance in Japan. In addition, the use of antiplatelet agents in the acute phase is essential, and issues regarding hemorrhagic complications remain to be addressed.

## Conclusions

Ruptured blood blister-like aneurysms (BBAs) of the basilar artery are rare and difficult to treat due to high rates of rebleeding and the presence of significant perforating branches. BBAs located on the posterior wall, in particular, are at high risk of ischemic complications. If coil embolization within the aneurysm is difficult, stenting alone may be an effective treatment, but it may be necessary to use two or more stents to achieve aneurysm occlusion. Treatment with a flow diverter is expected to be effective in the future.
